# Endocannabinoid-Mediated Control of Neural Circuit Excitability and Epileptic Seizures

**DOI:** 10.3389/fncir.2021.781113

**Published:** 2022-01-03

**Authors:** Yuki Sugaya, Masanobu Kano

**Affiliations:** ^1^Department of Neurophysiology, Graduate School of Medicine, The University of Tokyo, Tokyo, Japan; ^2^International Research Center for Neurointelligence (WPI-IRCN), The University of Tokyo Institutes for Advanced Study (UTIAS), The University of Tokyo, Tokyo, Japan

**Keywords:** endocannabinoid, 2-arachidonoylglycerol, CB_1_, CB_2_, epilepsy, seizure, cannabidiol

## Abstract

Research on endocannabinoid signaling has greatly advanced our understanding of how the excitability of neural circuits is controlled in health and disease. In general, endocannabinoid signaling at excitatory synapses suppresses excitability by inhibiting glutamate release, while that at inhibitory synapses promotes excitability by inhibiting GABA release, although there are some exceptions in genetically epileptic animal models. In the epileptic brain, the physiological distributions of endocannabinoid signaling molecules are disrupted during epileptogenesis, contributing to the occurrence of spontaneous seizures. However, it is still unknown how endocannabinoid signaling changes during seizures and how the redistribution of endocannabinoid signaling molecules proceeds during epileptogenesis. Recent development of cannabinoid sensors has enabled us to investigate endocannabinoid signaling in much greater spatial and temporal details than before. Application of cannabinoid sensors to epilepsy research has elucidated activity-dependent changes in endocannabinoid signaling during seizures. Furthermore, recent endocannabinoid research has paved the way for the clinical use of cannabidiol for the treatment of refractory epilepsy, such as Dravet syndrome, Lennox-Gastaut syndrome and tuberous sclerosis complex. Cannabidiol significantly reduces seizures and is considered to have comparable tolerability to conventional antiepileptic drugs. In this article, we introduce recent advances in research on the roles of endocannabinoid signaling in epileptic seizures and discuss future directions.

## Introduction

Epilepsy is one of the most common neurological disorders, with an incidence of 50.4 per 100,000 people per year (Ngugi et al., 2011). Although many drugs and surgical treatments are available for the treatment of epilepsy, approximately 30% of patients continue to have uncontrolled seizures despite treatment (Brodie et al., 2012). Therefore, there has been an urgent need to elucidate the etiology of epilepsy and to develop new treatments. Indeed, on both aspects of epilepsy research, significant progress has been made recently. In particular, the elucidation of the pathological mechanisms involving disruption of the endogenous cannabinoid (endocannabinoid) system has been advanced and therapeutic drugs targeting these mechanisms are developed, which is considered as a promising innovative therapeutic strategy for epilepsy. Previously, we reviewed findings up to 2018 on the epileptic seizures and endocannabinoid signaling (Sugaya and Kano, 2018). In this review article, we have assessed subsequent studies and revisited the role of endocannabinoid signaling in the control of excessive neuronal excitability and epileptic seizures.

## Endocannabinoids

Marijuana has been used for thousands of years for recreational and medical purposes, which led to extensive efforts to extract its active ingredients. Cannabidiol (CBD), which has become a subject of intense interest in recent years, was first successfully extracted in 1940 (Adams et al., 1940), and Δ-9-tetrahydrocannabinol (Δ^9^-THC), the main psychoactive component, in 1964 (Gaoni and Mechoulam, 1964). The G_i/o_ protein-coupled receptors for Δ^9^-THC, namely cannabinoid type 1 (CB_1_) and type 2 (CB_2_), were cloned in 1990 (Matsuda et al., 1990) and 1993 (Munro et al., 1993), respectively. The endogenous ligands for the cannabinoid receptors, namely, N-arachidonoyl ethanolamine (anandamide, AEA) and 2-arachidonoyl glycerol (2-AG), were identified in 1992 (Devane et al., 1992) and 1995 (Mechoulam et al., 1995; Sugiura et al., 1995), respectively. It has now been suggested that there are other receptors, besides CB_1_ and CB_2_, for these endocannabinoids, including G protein-coupled receptor 55 (GPR55) (Baker et al., 2006) and transient receptor potential vanilloid 1 (TRPV1) (Smart et al., 2000).

### Endocannabinoid Signaling Mediated by CB_1_ Receptor

**FIGURE 1:**
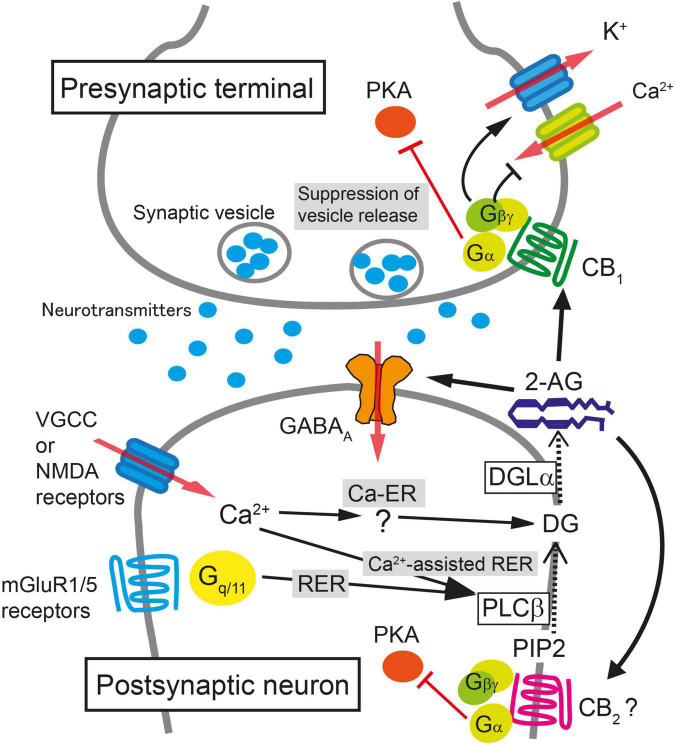
Schematic illustration of molecular mechanisms for endocannabinoid-mediated retrograde suppression of synaptic transmission. When intracellular Ca^2+^ concentration is elevated after the activation of voltage-gated Ca^2+^ channels (VGCC) or NMDA receptors, 2-AG is produced by diacylglycerol lipase α (DGLα) and released from postsynaptic neurons (Ca^2+^-driven endocannabinoid release; Ca-ER). The enzyme producing diacylglycerol (DG) in response to Ca^2+^ elevation has not been identified yet. When PLCβ is stimulated by the activation of mGluR1/5 or other G_q/11_-coupled receptors, DG is produced from phosphatidylinositol 4,5-bisphosphate (PIP2), DG is then converted to 2-AG by DGLα, and 2-AG is released from postsynaptic neurons (Receptor-driven endocannabinoid release; RER). When the activation of G_q/11_-coupled receptors and the elevation of intracellular Ca^2+^ concentration occur concurrently, the production of 2-AG is accelerated through PLCβ-dependent pathway (Ca^2+^-assisted receptor-driven endocannabinoid release; Ca^2+^-assisted RER). 2-AG released from postsynaptic neurons activates presynaptic CB_1_ receptors. 2-AG also activates CB_2_ receptors and is reported to potentiate agonist-mediated GABA_A_ receptor activation postsynaptically. Activation of CB_1_ receptor induces transient suppression of transmitter release through inhibition of VGCC and activation of K^+^ channels through Gβγ protein. Gα is responsible for the long-term suppression of transmitter release through inhibition of protein kinase A (PKA) signaling.

One of the most important discoveries in the field of cannabinoid research is the discovery that endocannabinoids function as a retrograde messenger at synapses—endocannabinoids mediate signals from depolarized postsynaptic neurons to presynaptic CB_1_ receptors, leading to a transient suppression of synaptic transmission (Kreitzer and Regehr, 2001; Ohno-Shosaku et al., 2001; Wilson and Nicoll, 2001). Furthermore, activation of G_q/11_ coupled-receptors in neurons releases endocannabinoids that retrogradely act on presynaptic CB_1_ receptors and induce transient synaptic suppression (Maejima et al., 2001; Varma et al., 2001). Soon after these discoveries, endocannabinoid-mediated long-term suppression of synaptic transmission was reported (Gerdeman et al., 2002; Marsicano et al., 2002; Robbe et al., 2002). Majority of short-term and long-term depression (STD and LTD, respectively) of synaptic transmission through the CB_1_ receptor is mediated by 2-AG (Kano et al., 2009; Luchicchi and Pistis, 2012). On the other hand, AEA is reported to be involved in LTD at certain types of synapses (Chávez et al., 2010; Khlaifia et al., 2013; Mathur et al., 2013). We will first describe CB_1_ receptor-dependent STD that is mediated by 2-AG (Figure 1), and then discuss the mechanisms of LTD that is mediated by 2-AG or AEA.

CB_1_ receptor-dependent STD occurs when a postsynaptic neuron is strongly depolarized and the intracellular Ca^2+^ level increases following Ca^2+^ influx through voltage-gated calcium channels. This STD of excitatory or inhibitory synaptic transmission is called depolarization-induced suppression of excitation (DSE) or inhibition (DSI), respectively. DSE/DSI was found to be totally abolished in mice deficient in diacylglycerol lipase α (DGLα), the major 2-AG producing enzyme from diacylglycerol (Bisogno et al., 2003), demonstrating that 2-AG is responsible for endocannabinoid-mediated STD (Gao et al., 2010; Tanimura et al., 2010). Biosynthesis of 2-AG induced by a large increase in the intracellular Ca^2+^ level alone is termed Ca^2+^-driven endocannabinoid release (Ca-ER) (Maejima et al., 2005; Hashimotodani et al., 2007a; Ohno-Shosaku et al., 2007). Another pathway for 2-AG production is initiated by the activation of G_q/11_ protein-coupled receptors, such as group I metabotropic glutamate receptors and M_1_/M_3_ muscarinic acetylcholine receptors, which is termed receptor-driven endocannabinoid release (RER) (Maejima et al., 2001; Fukudome et al., 2004; Hashimotodani et al., 2007a). Importantly, simultaneous weak activation of G_q/11_ protein-coupled receptors and small elevation of intracellular Ca^2+^ concentration effectively induces the production of 2-AG much larger than the 2-AG level by either stimulation alone, which is termed Ca^2+^-assisted receptor-driven endocannabinoid release (Ca-RER) (Hashimotodani et al., 2005, 2007a; Maejima et al., 2005). In postsynaptic neurons, the amount of 2-AG used for retrograde messengers might be regulated by the 2-AG-hydrolyzing enzymes α/β-hydrolase domain (ABHD) 6 and 12 (Blankman et al., 2007) and by a 2-AG oxidizing enzyme, cyclooxygenase (COX)-2 (Kozak et al., 2000). Due to its lipophilic nature, 2-AG is thought to pass through the plasma membrane, although it should cross the hydrophilic synaptic cleft toward presynaptic terminals; the details of these processes remain unknown.

After crossing the synaptic cleft, 2-AG binds to and activates CB_1_ receptors at the presynaptic terminal as a full agonist (Hillard, 2000). Activation of the CB_1_ receptor suppresses neurotransmitter release mainly by inhibiting voltage-gated calcium channels (Pertwee, 1997; Huang et al., 2001), activating G protein-gated inwardly rectifying potassium channel (Henry and Chavkin, 1995) and by suppressing the activity of the presynaptic protein Munc18-1 through the phosphorylation of extracellular-regulated kinase (Schmitz et al., 2016). The action of 2-AG is terminated when it is degraded by monoacylglycerol lipase (MGL) (Hashimotodani et al., 2007b; Zhong et al., 2011; Tanimura et al., 2012) or potentially by presynaptic COX-2 (Wang et al., 2005).

In addition to STD, presynaptic forms of 2-AG-mediated LTD have been reported in several brain regions. At inhibitory synapses in the hippocampus, the mechanisms of 2-AG production and release in LTD are similar to those in STD, but LTD induction requires 5 to 10 min of continuous CB_1_ receptor activation and presynaptic activity (Chevaleyre and Castillo, 2003). Activation of the CB_1_ receptor mobilizes G_α i/o_ protein and decreases cAMP production and protein kinase A (PKA) signaling, which eventually results in a persistent reduction in the efficacy of neurotransmitter release (Chevaleyre et al., 2007). It is noteworthy that the LTD is sustained without further activation of CB_1_ receptors after induction. Protein synthesis (Younts et al., 2016) as well as presynaptic proteins including RIM1α (Chevaleyre et al., 2007), calcineurin (Heifets et al., 2008), and potassium channels (Yasuda et al., 2008) are required for the induction of CB_1_-mediated LTD at inhibitory synapses in the hippocampus.

As described previously, the other major endocannabinoid AEA is crucial for the induction of CB_1_-mediated LTD at inhibitory synapses in the striatum (Mathur et al., 2013) and amygdala (Azad et al., 2004). Moreover, AEA-mediated LTD through TRPV1 was observed at perforant path–granule cell synapses in the dentate gyrus (Chávez et al., 2010) and in D_2_ dopamine receptor-positive medium spiny neurons in the nucleus accumbens (Grueter et al., 2010). Although the biochemical pathways for AEA synthesis are not fully understood, the cascade leading to the production of AEA involves the synthesis of N-acylphosphatidylethanolamine (NAPE) from phospholipids by N-acyltransferase (Cadas et al., 1997) and the degradation of NAPE by NAPE-specific phospholipase D to AEA (Okamoto et al., 2004; Leishman et al., 2016). AEA is cleaved to arachidonic acid by fatty acid amide hydrolase (FAAH) (Cravatt et al., 1996). AEA is also degraded by COX-2 in postsynaptic neurons (Yu et al., 1997).

In addition to the activity-dependent phasic suppression of synaptic transmission by 2-AG, endocannabinoid signaling through the CB_1_ receptor also mediates tonic suppression of synaptic transmission at inhibitory synapses in the CA3 (Losonczy et al., 2004) and CA1 (Neu et al., 2007; Lee et al., 2015) and at mossy cell–dentate granule cell excitatory synapses in the dentate gyrus (Jensen et al., 2021). The tonic CB_1_ signaling could be due to the continuous release of endocannabinoids (Neu et al., 2007; Lee et al., 2015) or constitutively active CB_1_ receptors (Lee et al., 2015; Jensen et al., 2021).

Detailed mechanisms of endocannabinoid-mediated regulation of synaptic transmission through the CB_1_ receptor have been described in several excellent reviews (Heifets and Castillo, 2009; Kano et al., 2009; Castillo et al., 2012).

### CB_2_ Receptor-Mediated Signaling

The CB_2_ receptor is the other canonical G_i/o_-coupled cannabinoid receptor that mediates endocannabinoid signaling (Munro et al., 1993). In the central nervous system, the expression of CB_2_ receptor mRNA in neurons has been confirmed using fluorescent *in situ* hybridization (Li and Kim, 2015; Stempel et al., 2016). Unfortunately, the CB_2_ receptor protein expression in neurons remains elusive because of the lack of specific CB_2_ receptor antibodies. However, recent studies using CB_2_ knockout mice demonstrated that CB_2_ receptors contribute to the regulation of neuronal excitability and synaptic transmission. CB_2_ receptor signaling is required for maintaining excitatory synaptic transmission and LTP at Schaffer collateral–CA1 pyramidal cell synapses (Li and Kim, 2016). In contrast, CB_2_ receptor signaling has a long-lasting hyperpolarizing effect in hippocampal CA3 pyramidal cells (Stempel et al., 2016). Long-lasting hyperpolarization mediated by CB_2_ receptor signaling has also been reported in layer 2/3 regular spiking non-pyramidal cells in the somatosensory cortex (Stumpf et al., 2018). Furthermore, firing of dopamine neurons in the ventral tegmental area has been reported to decrease through activation of CB_2_ receptors *in vivo* (Zhang et al., 2014). Thus, the effects of CB_2_ receptor signaling on neuronal network activity differ depending on brain areas and cell types.

### Endocannabinoid Imaging

In most studies, endocannabinoid levels in the brain have been measured by using chromatography and mass spectrometry, which cannot provide information about detailed spatiotemporal dynamics of endocannabinoid release. Recently, there has been a breakthrough in this research field. Dong et al. (2021) developed a genetically encoded tool for sensing endocannabinoids based on the human CB_1_ receptor and circular-permutated GFP, named GRABeCB2.0. The range of fluorescence change of this protein is sufficient for the detection of physiological endocannabinoid concentrations, and has a rise time and decay time of 1.6 and 11.2 s, respectively (Dong et al., 2021). Furthermore, the GRABeCB2.0 sensor binds endocannabinoids such as 2-AG and AEA, but does not couple to the downstream G proteins and has no observable effects on cellular physiology (Dong et al., 2021). These features enable us to use this sensor to observe physiological endocannabinoid release in cultures, brain slices and the brain of live animals during behavioral experiments (Dong et al., 2021; Farrell et al., 2021). For example, spontaneous running induces an increase in the signal for 2-AG release in the pyramidal cell layer of the hippocampal CA1 in mice (Farrell et al., 2021). Moreover, the increase was correlated with the increase in cellular activity observed using the calcium sensor jRGECO1a. These results indicate the presence of activity-dependent 2-AG release in the brain of living animals. As described later, the sensor was also used to detect a transient surge of 2-AG release during seizures *in vivo* (Farrell et al., 2021).

## Endocannabinoid Signaling and Epilepsy

In this section, we will discuss previous findings related to endocannabinoid signaling in patients with epilepsy, while the findings in animal models of chronic epilepsy and acute seizures will be discussed in later sections. Brains of patients with mesial temporal lobe epilepsy (mTLE) with hippocampal sclerosis show reduced levels of DGLα mRNA compared to specimens from the non-epileptic control subjects (Ludányi et al., 2008). In contrast, there were no differences between control and epileptic patients in terms of the mRNA levels of NAPE-specific phospholipase D, MGL, or FAAH (Ludányi et al., 2008). Therefore, 2-AG production seems to be reduced, while 2-AG degradation, AEA production, and AEA degradation appear to be normal in the hippocampi of mTLE patients. A recent study on the concentration of endocannabinoids in brain samples from mTLE patients revealed that the 2-AG level was decreased to 51 and 65% in the hippocampus and the temporal cortex, respectively, of that in respective brain area of the autopsy control group (Rocha et al., 2020). The AEA concentration was unchanged in the hippocampus but increased to 175% in the temporal cortex compared to that of the autopsy control group (Rocha et al., 2020). These findings are consistent with the changes in mRNA levels in the hippocampus of mTLE patients (Ludányi et al., 2008), indicating a significant decrease in the 2-AG levels in epileptic foci. In contrast, another study reported that the concentration of AEA in the cerebrospinal fluid is reduced in untreated mTLE patients, whereas the level of 2-AG was similar between control subjects and mTLE patients (Romigi et al., 2010). It should be noted that the concentrations of 2-AG and AEA in the cerebrospinal fluid were measured during the inter-ictal state, it is therefore possible that the concentrations of 2-AG and AEA around the epileptic focus might be different.

The expression levels of CB_1_ and CB_2_ receptors have also been investigated. The mRNA level of the CB_1_ receptor in the hippocampus was significantly decreased in patients with mTLE when compared to that in control subjects (Ludányi et al., 2008). This decreased expression was more pronounced in mTLE patients with hippocampal sclerosis, suggesting a negative correlation between the mRNA level of the CB_1_ receptor and disease progression. Electron microscopic analysis revealed the loss of CB_1_ receptor protein in excitatory axon terminals, as well as a decrease in the total number of excitatory synapses in the inner molecular layer of the dentate gyrus of mTLE patients (Ludányi et al., 2008). In contrast, the expression levels of CB_1_ receptors at inhibitory axon terminals and the number of inhibitory axon terminals in the dentate gyrus of mTLE patients were comparable (Ludányi et al., 2008) or even increased (Maglóczky et al., 2010) compared to those of control subjects. As mentioned before, CB_1_ receptor signaling suppress synaptic transmission at excitatory and inhibitory synapses. Consequently, decrease in CB_1_ receptor signaling at excitatory synapses potentially results in excessive excitatory input, whereas its increase at inhibitory synapses causes disinhibition of postsynaptic neurons in a tonic and phasic manner. Therefore, changes in endocannabinoid signaling molecules at both excitatory and inhibitory synapses observed in aforementioned studies can potentially increase the excitability of the dentate gyrus, which may underlie the chronic susceptibility to seizures seen in patients with epilepsy.

In the hippocampus proper, an increase in the expression of CB_1_ receptors was observed in the stratum oriens of the area CA2 and CA3 in mTLE patients with hippocampal sclerosis (Ludányi et al., 2008). Consistent with the results of immunohistochemical staining, a study using positron emission tomography (PET) showed that the availability of CB_1_ receptor in the ipsilateral temporal lobe was increased in mTLE patients with hippocampal sclerosis when compared to that in healthy volunteers (Goffin et al., 2011). Since this study used a high affinity PET tracer, [^18^F]-MK-9470 (Burns et al., 2007), the increase in the availability of CB_1_ receptor is considered to reflect the increase in the expression of the receptor. In a recent study, the degree of CB_1_ and CB_2_ activation after the application of the CB_1_ agonist WIN55212-2 was investigated using the [^35^S] GTPγS binding assay in human brain samples. The authors found that WIN55212-2 induced higher [^35^S] GTPγS binding, suggesting higher G_*i/o*_ protein activation in the hippocampus of mTLE patients (Rocha et al., 2020). Furthermore, the authors performed a binding assay with CB_1_ and CB_2_ receptor blockers (AM251 and AM630, respectively; 100 μM) and showed that CB_1_ receptors mediated the higher G_*i/o*_ protein activation induced by WIN 55212-2 in the hippocampus of mTLE patients (Rocha et al., 2020). These results are consistent with the higher expression of CB_1_ receptors in the hippocampus of patients with mTLE, as described above (Ludányi et al., 2008; Goffin et al., 2011). The cell types responsible for the increased expression of CB_1_ receptors in the stratum oriens of the hippocampus in epileptic patients are still unknown. However, as described later, increased CB_1_ receptor expression in inhibitory neurons of the hippocampal CA1 has been demonstrated in animal models of epilepsy (Chen et al., 2003; Maglóczky et al., 2010). Therefore, the increase in CB_1_ receptor expression in the hippocampus of human epileptic patients may originate from inhibitory neurons, which may cause disinhibition.

While CB_1_ receptor expression is hardly observed in astrocytes in non-epileptic control subjects, it was found in astrocytes of over 50% of the hippocampal specimens taken from epileptic patients (Meng et al., 2014). Activation of CB_1_ receptors in astrocytes is reported to promote glutamate release from these cells in mice (Han et al., 2012), suggesting that the expression of CB_1_ receptors in hippocampal astrocytes of mTLE patients increases the excitability of surrounding neural circuits. However, the effect of the increased expression of CB_1_ receptors in astrocytes can be counterbalanced by increased astrocytic expression of the endocannabinoid degradation enzyme COX-2 in epileptic patients (Desjardins et al., 2003).

In several studies, mutations in genes of endocannabinoid signaling molecules in human epileptic patients have been investigated intensively. The details have been described in our previous review article (Sugaya and Kano, 2018).

## Epileptogenesis

It is known that epileptic patients frequently have experience of brain insult such as febrile seizure, central nervous system infection, and traumatic brain injury (TBI) several years before the onset of recurrent spontaneous seizures. The period between the initial insult and the onset of spontaneous seizures is called the latent period. The discovery of kindling (Goddard, 1967), i.e., repeated intermittent stimulation of neural circuits resulting in progressive intensification of seizure responses to stimuli, led to the hypothesis that anatomical and physiological changes gradually progress during the latent period and result in the development of epileptic foci from which seizures arise. This process is called epileptogenesis. On the other hand, the process that takes place during the transition from the non-seizure state to seizure state is called ictogenesis.

**FIGURE 2:**
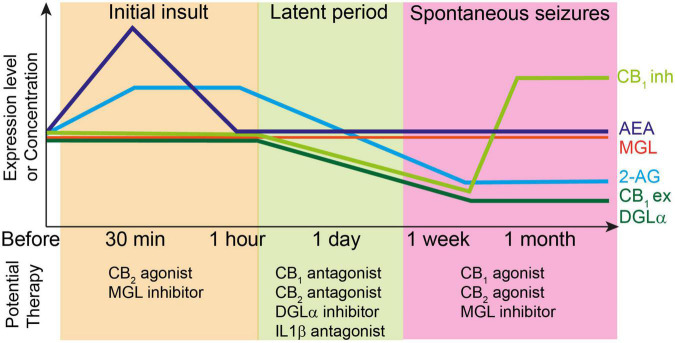
Top: Changes in the levels of endocannabinoids and molecules related to endocannabinoid signaling during epileptogenesis. Each line represents relative changes from the level before the initial insult such as status epilepticus and febrile seizures. CB_1_ex and CB_1_inh indicate expression level of CB_1_ receptors at excitatory and inhibitory synapses, respectively. Initial insult increases the 2-AG and AEA levels, which induces the subsequent decrease in the expression levels of several endocannabinoid-related molecules such as DGLα and CB_1_ receptors during latent period. After the onset of spontaneous seizures, the expression level of CB_1_ receptors at excitatory synapses and that of DGLα remain decreased, whereas that of CB_1_ receptors at inhibitory synapses increases beyond the level before initial insult. The 2-AG level is also decreased presumably due to the decreased expression of DGLα. Bottom: potential therapy for the prevention of epileptogenesis or seizures at each stage of epileptogenesis.

To investigate epileptogenesis, severe initial insults, such as prolonged generalized tonic-clonic seizures that is termed status epilepticus (SE), febrile seizures in newborns, and TBI are delivered to animals. These severe insults trigger epileptogenesis in neural circuits and result in spontaneous seizures several days to weeks after the insults (Figure 2). Pilocarpine, an agonist of muscarinic acetylcholine receptors, or kainate, an agonist of the kainate-type as well as AMPA-type ionotropic glutamate receptors, are often administered for the induction of SE. Kindling has also been frequently used for the study of epileptogenesis. The advantage of kindling is that it enables the observation of the step-by-step processes of epileptogenesis. Electrical stimulation of the lateral amygdala or perforant path are common kindling models of mTLE. Other protocols for kindling include electrical corneal stimulation and chemical kindling using GABA_A_ receptor antagonists such as pentylenetetrazole (PTZ) or picrotoxin. All kindling protocols fulfill the famous notion of “seizures beget seizures,” which means that seizures promote epileptogenesis.

### Involvement of 2-AG and AEA

When the concentration of 2-AG was measured in rat hippocampi 15 min after the initiation of pilocarpine-induced SE, an approximately 1.5-fold increase in 2-AG levels compared to vehicle-treated controls was observed (Wallace et al., 2003). Similarly, the 2-AG level in brain tissues 100 min after kainate treatment in 8-week-old rats was 1.5-fold higher than that in vehicle-treated controls (Fezza et al., 2014). Recently, the endocannabinoid sensor GRABeCB2.0 has been developed (Dong et al., 2021). By imaging fluorescent signals representing released endocannabinoids by using GRABeCB2.0, a transient surge of endocannabinoid release was successfully detected during afterdischarges evoked by the electrical stimulation of the contralateral hippocampus (Farrell et al., 2021). Importantly, the authors’ pharmacological examination demonstrated clearly that 2-AG, but not AEA, is produced during afterdischarges.

However, in sharp contrast to the above results, Marsicano et al. (2003) reported that the concentration of 2-AG in the hippocampi of adult mice 20 min after kainate injection was not different from the pre-treatment level. Instead, the concentration of AEA increased significantly to up to 300% of the pre-treatment level and decreased back to baseline within 1 h after kainate treatment (Marsicano et al., 2003). These results are consistent with a recent study showing no change in the levels of 2-AG and AEA in the cerebral cortex, striatum, hippocampus, thalamus, hypothalamus, and cerebellum 1 h after kainate injection (Lerner et al., 2017). In another study using the kindling model, there were no changes in 2-AG and AEA levels in the hippocampus 4 weeks after PTZ kindling (Hansen et al., 2009).

The reason for these discrepant results of 2-AG and AEA levels could be the difference in the timing of sampling of brain tissues after SE or kindling epileptogenesis. As observed in the endocannabinoid imaging using GRABeCB2.0, the endocannabinoid level changes rapidly depending on neural activity, which is ascribed to rapid degradation of endocannabinoids (Farrell et al., 2021). In addition, SE occurs intermittently after kainate or pilocarpine injection. Therefore, endocannabinoid levels could differ significantly depending on the timing of sampling of brain tissues. It is possible that elevation of endocannabinoid concentration after seizures was not detected if sampling of brain tissues was performed during inter-ictal periods or after mild seizures.

Changes in the expression of enzymes related to endocannabinoid synthesis and degradation have also been investigated (Figure 2). We investigated the expression of DGLα in the hippocampal CA1 and dentate gyrus of mice that received unilateral intrahippocampal kainate injection and reported a significant decrease in the expression of DGLα 2 weeks after kainate injection (Sugaya et al., 2016). The decreased DGLα expression in the hippocampi of epileptic mice is consistent with that in the hippocampi of patients with mTLE (Ludányi et al., 2008). In contrast, the level of MGL mRNA was unchanged 1 h and 7 days after kainate-induced SE (Terrone et al., 2018). These results suggest that production of 2-AG is decreased in epileptic brains. Moreover, decreased expression of DGLα may affect the process of epileptogenesis (Table 1). We demonstrated that 2-AG prevented epileptogenesis in a perforant path kindling model, and that loss of 2-AG promoted the kindling process (Sugaya et al., 2016). These data are consistent with the results obtained in amygdala-based (von Rüden et al., 2015a) and corneal (Griebel et al., 2015) kindling models. The 2-AG-mediated anti-epileptogenic effect could be due to the preventive effect of 2-AG against neuronal injury. Preventive effects of MGL inhibitors against neuronal degeneration *in vitro* (Kallendrusch et al., 2012) and in a kainate-induced SE model *in vivo* (Terrone et al., 2018) have also been reported. The MGL inhibitor CPD-4645 also decreased the mRNA level of the inflammation marker interleukin-1β at 1 h and at 7 days after kainate-induced SE (Terrone et al., 2018).

**TABLE 1:** Epileptogenesis and ictogenesis modulated by manipulations of endocannabinoid signaling.

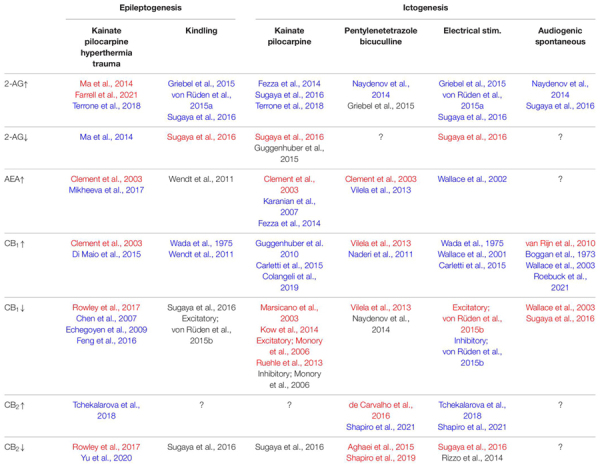

*Summary of the effects of enhancing or suppressing endocannabinoid signaling (2-AG, AEA, CB_1_ or CB_2_) on epileptogenesis and ictogenesis in various rodent seizure models.*

*Cells in the leftmost column indicate the manipulations of endocannabinoid signaling molecules and those in the first and second rows show rodent seizure models.*

*The observed effect in each study was represented as follows; blue, suppression of epileptogenesis or ictogenesis; black, no change; red, promotion of epileptogenesis or ictogenesis; ?, unknown.*

*Excitatory, results observed in mice with excitatory neuron specific deletion of CB_1_ receptors; Inhibitory, results observed in mice with inhibitory neuron specific deletion of CB_1_ receptor.*

However, in sharp contrast to the results mentioned above, DGLα blockade by RHC80267 for 7 days initiated immediately after the termination of pilocarpine-induced SE decreased cell death and the frequency of spontaneous seizures (Ma et al., 2014), indicating that 2-AG promotes SE-induced epileptogenesis. The pro-epileptogenic effect of 2-AG during the initial 7 days after SE may be explained by the downregulation of CB_1_ receptor signaling. Considering the fact that 5 days of 2-AG upregulation by treatment with the MGL inhibitor JZL 184 [16 mg/kg, intraperitoneal (i.p.)] induced the downregulation of CB_1_ receptor signaling in non-epileptic wild-type mice (Kinsey et al., 2013), it is likely that downregulation of CB_1_ receptor signaling at excitatory synapses occurs after sustained increase in 2-AG levels after SE. Another potential explanation for the pro-epileptogenic effects of 2-AG would be prostaglandin-mediated stroke-like hypoperfusion events that occur after seizures (Farrell et al., 2021). The hydrolysis of 2-AG by MGL produces arachidonic acid which is broken down to vasoactive prostaglandins by COX-2. Therefore, surge of 2-AG production during seizures results in the vasoconstriction and hypoxia around the seizure focus for tens of minutes (Farrell et al., 2021), which could potentially damage the tissues around the seizure focus and promote epileptogenesis. Taken together, it is thought that the 2-AG level initially increases during SE and then decreases along with the decreased expression of DGLα in the chronic phase of epileptogenesis. The role of 2-AG in epileptogenesis may differ depending on the level and the duration of 2-AG surge.

In contrast to the aforementioned effects of 2-AG, increase in the level of AEA by treatment with the FAAH inhibitor URB597 did not affect kindling epileptogenesis (Wendt et al., 2011). As for SE-induced epileptogenesis, elevation of the AEA level is reported to exert different effects depending on the degree of elevation (Table 1)—a mild increase prevented (Mikheeva et al., 2017) whereas a strong increase promoted cell death (Clement et al., 2003).

### Involvement of CB_1_ and CB_2_ Receptors

It has also been reported that, as with DGLα, expression of the CB_1_ receptor is affected by epileptogenesis (Figure 2). In a PET imaging study on rhesus monkeys during kindling epileptogenesis, changes in CB_1_ receptor availability and in glucose metabolism were observed using [^18^F]-MK-9470 and [^18^F]-FDG, respectively (Cleeren et al., 2018). Since [^18^F]-MK-9470 is a high affinity PET tracer of CB_1_ receptor (Burns et al., 2007), its signal is thought to reflect the expression level of CB_1_ receptor. The results of the PET study suggested that CB_1_ receptor expression around the kindling electrode in the right amygdala and the ipsilateral thalamus increased from the initial stage of kindling and further increased gradually throughout kindling. Moreover, in the ipsilateral insula, the number of voxels showing decreased CB_1_ receptor expression gradually increased throughout kindling. The finding of increased CB_1_ receptor expression around the epileptic focus in the epileptic brain of primates using PET imaging is consistent with the findings in humans (Goffin et al., 2011) as described above.

In the cerebral cortex and the hippocampus, CB_1_ receptors are found in axon terminals of cholecystokinin (CCK)-positive interneurons and those of pyramidal neurons (Marsicano and Lutz, 1999). Wyeth et al. (2010) reported that during pilocarpine induced epileptogenesis in mice, CCK-labeled boutons were degenerated and CB_1_ receptor expression was decreased, whereas parvalbumin-containing boutons were preserved in the pyramidal cell layer of CA1. The decrease in CB_1_ receptor expression was observed from 48 h after SE. In the strata oriens and radiatum of CA1, however, the level of CCK labeling was initially decreased but then recovered and even increased compared to the initial level at one and 2 months after pilocarpine-induced SE. Notably, CB_1_ receptor expression remained low in those layers 2 months after pilocarpine-induced SE. These results suggest that CB_1_-dependent regulation of inhibitory synaptic transmission in CCK-positive interneurons is lost in the pyramidal cell layer but is preserved in the strata oriens and radiatum in CA1 during chronic epilepsy. A pronounced loss of CB_1_ receptor expression was also observed in the entire hippocampus of mice that experienced SE-induced epileptogenesis for shorter than 2 weeks in kainate model (Sugaya et al., 2016). In rats, pilocarpine-induced SE was found to induce a time-dependent redistribution of CB_1_ receptors in the hippocampus (Falenski et al., 2007, 2009). Within 1 week of SE, CB_1_ receptor expression was lost throughout the hippocampus, followed by restoration of CB_1_ receptor expression in the CA1, but not in the dentate gyrus, 2 weeks after SE. Then, a characteristic redistribution of CB_1_ receptors followed in the hippocampus 1 month after SE. Namely, CB_1_ receptor immunoreactivity decreased in the dentate gyrus inner molecular layer and the CA1 pyramidal cell layer and, conversely, it increased in the strata oriens and radiatum of the CA1–3 (Falenski et al., 2009). This unique redistribution of CB_1_ receptors was sustained for up to 6 months in chronic epileptic rats. The pattern of redistribution is consistent with the distribution of CB_1_ receptors in the hippocampi of chronic mTLE patients (Ludányi et al., 2008; Maglóczky et al., 2010). Since spontaneous seizures are observed at 2 weeks after SE in mice (Sugaya et al., 2016) and rats (Goffin et al., 2007; Williams et al., 2009), the loss of CB_1_ receptors in the inner molecular layer of the dentate gyrus and in the hippocampal CA1 are likely to be the primary cause of the occurrence of spontaneous seizures. On the other hand, the subsequent increase of CB_1_ receptors in the strata radiatum of the CA1–3 could be originated from inhibitory synapses (Maglóczky et al., 2010). It is still unknown whether the observed increase of CB_1_ receptor expression in these areas is due to increase in the CB_1_ receptor density at each inhibitory synaptic terminal or increase in the number of CB_1_-positive inhibitory terminals without changes in the level of CB_1_ receptor expression at each terminal. In the former case, enhanced endocannabinoid-mediated reduction of GABA release would cause disinhibition of these regions and promote the disease progression. In the latter case, sprouting of the axons of CB_1_-positive GABAergic interneurons and consequent increase in the number of GABAergic terminals might elevate GABA release and suppress spontaneous seizures.

Changes in CB_1_ receptor expression were also observed in a different model of epileptogenesis. Chen et al. (2003) used a rat febrile seizure model in which the body temperature of rat pups was raised to 41–42°C to evoke seizures for about 20 min on postnatal day 10. This is an animal model of human complex febrile seizures that increase the risk of developing mTLE later in life. The authors found that the febrile seizures induced a significant increase in the expression of CB_1_ receptors in CCK-positive interneurons, resulting in persistent enhancement of DSI in hippocampal CA1 pyramidal neurons for up to 5 weeks (Chen et al., 2003). In contrast, no significant effect was observed on DSE in CA1 pyramidal neurons at 5 weeks after the febrile seizures (Chen et al., 2003). These results suggest that neonatal febrile seizures change the expression pattern of CB_1_ receptors in the hippocampus during development and promote disinhibition of hippocampal neural circuits, which might contribute to epileptogenesis. Importantly, a single intraperitoneal injection of the CB_1_ antagonist SR141716A (1 mg/kg, i.p.) into rat pups 1 h before the start of febrile seizures blocked the seizure-induced enhancement of DSI and increase of CB_1_ receptors in adults (Chen et al., 2007).

Taken together, these results indicate that CB_1_ receptor expression increases in the later stage of SE- or febrile seizure-induced epileptogenesis, which can be blocked by CB_1_ receptor antagonists before febrile seizures. Therefore, intense activation of CB_1_ receptors at the time of initial insult might trigger processes for the increase in CB_1_ receptor expression in the later stages. Detailed molecular mechanisms underlying these phenomena remain elusive, although inflammation and subsequent cytokine release could be involved. In one study in rats, interleukin-1β level was found to be elevated until 12 h after febrile seizures, and an antagonist of the interleukin-1β receptor prevented the increase in CB_1_ receptor expression after febrile seizures (Feng et al., 2016).

The risk of epilepsy is reported to increase by two to seven folds after TBI (Christensen et al., 2009). In a rat model of TBI-induced epilepsy, treatment with the CB_1_ antagonist SR141716A immediately after cerebral cortex injury prevented long-term increase in seizure susceptibility (Echegoyen et al., 2009). This result, taken together with the findings from the febrile seizure model, suggests that short-term blockade of CB_1_ receptor signaling immediately after the initial insult can be broadly applicable as a preventive strategy for epileptogenesis (Figure 2 and Table 1). It is important to note that the blockade of CB_1_ receptor signaling may have to be finished within a day because the treatment with a CB_1_ agonist, WIN55212-2, for 2 weeks starting 1 day after pilocarpine-induced SE significantly reduce the number of seizures observed 1 to 6 months after SE (Di Maio et al., 2015).

Consistent with the change in the later stages of SE- or febrile seizure-induced epileptogenesis, CB_1_ receptor expression was found to be increased in the hippocampal CA1 and dentate gyrus after amygdala kindling in mice (von Rüden et al., 2015b). The role of CB_1_ receptors in kindling epileptogenesis was investigated using knockout mice and pharmacological interventions (Table 1). The results showed that there was no difference between the mice with conditional CB_1_ knockout in excitatory neurons and their control littermates with regard to the speed of kindling epileptogenesis (von Rüden et al., 2015b), which suggests that the lack of CB_1_ receptor signaling in excitatory neurons does not affect kindling epileptogenesis. Furthermore, similar results were obtained for CB_1_ receptor signaling in inhibitory neurons (von Rüden et al., 2015b). These findings are in sharp contrast with the results obtained from DGLα knockout mice (Sugaya et al., 2016), from wild-type mice treated with WIN55212-2 (Wendt et al., 2011) and from wild-type mice with 2-AG augmentation (Griebel et al., 2015; von Rüden et al., 2015a; Sugaya et al., 2016), which have consistently reported anti-epileptogenic effects of endocannabinoid signaling in kindling.

Reasons for the phenotypic differences between these studies remain unclear. However, the involvement of CB_2_ receptors in kindling development could explain this contradiction (Sugaya et al., 2016). We have reported that the blockade of both CB_1_ and CB_2_ receptors significantly promoted kindling, as was observed in DGLα knockout mice (Sugaya et al., 2016). A subsequent study demonstrated the occurrence of spontaneous seizures in CB_1_ and CB_2_ double knockout mice (Rowley et al., 2017). Importantly, spontaneous seizures were not observed in CB_1_ or CB_2_ single knockout mice, indicating the complementary roles played by CB_1_ and CB_2_ receptors in the prevention of epileptogenesis (Rowley et al., 2017). Recently, several researchers investigated the role of CB_2_ receptor signaling in epileptogenesis in more detail. In contrast to the decrease in the expression of CB_1_ receptor mRNA, that of CB_2_ receptor mRNA tended to increase after kainate-induced SE (Yu et al., 2020). Moreover, the CB_2_ receptor agonist β-caryophyllene was reported to reduce the severity of kainate-induced seizures when it was chronically administered prior to kainate injection in wild-type mice (Tchekalarova et al., 2018), indicating that CB_2_ receptor signaling is anti-epileptogenic. Interestingly, transient inverse agonism of CB_2_ receptor signaling by SMM-189 2 h after the start of SE significantly decreased neuronal degeneration in the CA1 and suppressed brain inflammation, as assessed based on the levels of interleukin-1β, interleukin-6, tumor necrosis factor α, chemokine (C-C motif) ligand 2 (CCL2), CCL3, and CCL4 (Yu et al., 2020). These results support the idea that transient suppression of CB_2_ receptor signaling immediately after the initial insult may prevent epileptogenesis, which is analogous to the observations for the CB_1_ receptor. Taken together, it is considered that, similar to CB_1,_ CB_2_ receptor signaling can prevent or promote epileptogenesis depending on the timing of intervention (Figure 2 and Table 1). Further studies using conditional CB_2_ receptor knockout mice will be required to elucidate the precise role of this receptor in epileptogenesis.

In many genetic models of epilepsy, it has been shown that genetic mutations cause neural circuit hyperexcitability, which in turn affects the expression of CB_1_ receptors. For example, absence seizures, a type of seizure involving thalamo-cortical circuits, are present in the WAG/Rij rat strain (van Luijtelaar and Coenen, 1986; Coenen et al., 1992) and Genetic Absence Epilepsy Rats from Strasbourg (GAERS) (Vergnes et al., 1982). Electroencephalography (EEG) experiments revealed that these strains of rats showed frequent spike-and-wave discharges (Vergnes et al., 1982; Coenen et al., 1992), which is the typical EEG pattern observed in patients during absence epilepsy. Western blotting-based analysis of WAG/Rij rats (van Rijn et al., 2010) indicated that CB_1_ receptor expression decreased to about 50% in the reticular thalamic nucleus and 70% in the ventrobasal thalamic nuclei at the age of 8 months compared to that in age-matched August Copenhagen Irish (ACI) rats (van Rijn et al., 2010). Interestingly, the expression of CB_1_ receptor mRNA quantified by using *in situ* hybridization was significantly decreased in 8 month old but not 2 month old WAG/Rij rats compared to that in age-matched ACI rats (van Rijn et al., 2010), suggesting that the change in CB_1_ receptor reflects the pathological progression of absence seizures. On the other hand, the decrease in CB_1_ receptor expression was observed in the cortex and the hippocampus, but not in the thalamus of GAERS rats compared to control rats (Roebuck et al., 2021). In a mouse model of Dravet syndrome wherein the mice carry a missense mutation (A1783V) in the *Scn1a* gene that encodes the α subunit of a voltage-gated sodium channel, decreased CB_1_ but increased CB_2_ receptor expression was reported in the hippocampus by western blotting analysis (Satta et al., 2020). In contrast, in Wister audiogenic rats, CB_1_ receptor expression in the hippocampus and amygdala was found to be increased after acute and chronic audiogenic seizures induced by sound stimulation (110–120 dB, 5–20 kHz, 60 s maximum) (Lazarini-Lopes et al., 2020). Taken together, these results indicate that the pattern of CB_1_ expression and its changes with age or in response to seizures differ greatly in different genetically epileptic rodent models. Further studies are needed to elucidate the roles of CB_1_ receptor signaling in epileptogenesis in respective rodent models.

## Ictogenesis

As described above, ictogenesis is the process of transition from the non-ictal (non-seizure) to ictal (seizure) state. This process is usually investigated in epileptic animals showing spontaneous seizures or in non-epileptic animals subjected to chemical convulsants or electrical stimulation (Figure 2 and Table 1).

### Involvement of 2-AG

The role of 2-AG signaling in ictogenesis has been investigated using DGLα knockout mice (Sugaya et al., 2016). After kainate injection (30 mg/kg, i.p.), DGLα knockout mice showed shorter latency to tonic-clonic seizures and a higher mortality rate than wild-type mice. In addition, DGLα knockout mice exhibited longer afterdischarges in the dentate gyrus than wild-type mice when the perforant path was electrically stimulated. Consistently, MGL inhibitor, JZL184, ameliorated kainate-induced SE (Fezza et al., 2014; Sugaya et al., 2016). These results suggest that 2-AG is crucial for the suppression of seizures in the hippocampus.

A contrasting result was reported in the excitatory neurons of the dentate hilus, CA1, and CA3 of MGL-overexpressing mice (Guggenhuber et al., 2015). In pyramidal neurons of these mice, DSE was abolished, whereas DSI was intact, possibly because of the immediate degradation of 2-AG at excitatory synapses. However, no differences were observed in the severity of kainate-induced seizures (35 mg/kg, i.p.) compared to those in control mice, which is contrast to aforementioned results in DGLα knockout mice (Sugaya et al., 2016). The difference in seizure susceptibility between these two mouse lines could be ascribed to the difference in the concentration of 2-AG in the hippocampal tissues. In the hippocampi of MGL-overexpressing mice, the level of 2-AG was approximately half that in control mice, while that in DGLα knockout mice was reduced to approximately 1/10 of that in wild-type mice (Tanimura et al., 2010). Furthermore, DSE requires a much higher 2-AG level than DSI (Ohno-Shosaku et al., 2002). Therefore, even in the absence of DSE in the hippocampus of MGL-overexpressing mice, the occurrence of kainate-induced seizures might be suppressed by the remaining level of 2-AG. Another possibility would be that the kainate-induced seizures, which lasted for more than 3 h in the study, resulted in 2-AG production while a few seconds of depolarization failed to produce sufficient 2-AG for DSE. This prolonged 2-AG synthesis might have overwhelmed the capacity of 2-AG degradation by the overexpressed MGL.

As expected, based on the crucial role of 2-AG in the prevention of kainate-induced seizures, augmentation of 2-AG concentration over the physiological level has been reported to ameliorate seizures (Table 1). Administration of an MGL inhibitor to wild-type mice reduced the severity of various types of evoked seizures (Griebel et al., 2015; von Rüden et al., 2015a; Sugaya et al., 2016). Moreover, a recent study using the MGL inhibitor CPD-4645 demonstrated that the ameliorating effect of MGL inhibition on kainate-induced SE involved 2-AG-mediated activation of the CB_1_ receptor initially, but later was predominantly mediated through mechanisms independent of CB_1_ receptor signaling (Terrone et al., 2018), which is consistent with the potential role of the CB_2_ receptor in seizure protection.

WWL123, an inhibitor of the postsynaptic 2-AG degrading enzyme ABHD6, is also capable of reducing the severity of PTZ-induced seizures (Naydenov et al., 2014). Surprisingly, the suppressive effect of WWL123 on PTZ seizures was observed in CB_1_ and CB_2_ double knockout mice and therefore is thought to be independent of the CB_1_ and CB_2_ receptors. The primary target of increased 2-AG at postsynaptic neurons could be GABA_A_ receptors, as 2-AG directly activated them (Naydenov et al., 2014). In contrast, the MGL inhibitor SAR127303 did not change the minimal dose of PTZ required to induce seizures (Griebel et al., 2015), suggesting that postsynaptic increase in 2-AG is important.

These results demonstrate the suppressive effect of 2-AG on seizures in the non-epileptic brain (Table 1). In parallel, the role of 2-AG signaling in spontaneous seizures in the epileptic brain has also been investigated (Table 1). We have reported that the MGL inhibitor JZL184 has a suppressive effect on the frequency of spontaneous seizures after kainate-induced SE (Sugaya et al., 2016). In genetically epileptic mice, pharmacological blockade of ABHD6 by means of WWL123 completely suppressed the occurrence of spontaneous seizures in the R6/2 mouse strain (Naydenov et al., 2014). These results suggest that augmentation of 2-AG signaling strongly suppresses spontaneous seizures in the epileptic brain (Figure 2 and Table 1).

### Involvement of AEA

Direct application of AEA or augmentation of AEA levels by blocking its degradation using an FAAH inhibitor suppresses the seizures induced by transcorneal electrical stimulation (Wallace et al., 2002). Since such a seizure-suppressive effect was abolished by co-administration of the CB_1_ antagonist SR141716A, the suppressive effect of AEA on evoked seizures was thought to be mediated by the CB_1_ receptor. FAAH inhibitors also suppress seizures induced by kainate (Karanian et al., 2007; Fezza et al., 2014) and PTZ (Vilela et al., 2013; Table 1).

However, contradictory results were obtained in a study using FAAH knockout mice. AEA levels in the cortex, hippocampus, and cerebellum were 10 times higher in FAAH knockout mice than in wild-type mice, presumably because of the slower degradation of AEA. However, kainate-induced seizures were more severe in FAAH knockout mice than in wild-type mice (Clement et al., 2003). Moreover, administration of AEA in FAAH knockout mice further aggravated the kainate-induced seizures and cell death. A CB_1_ blocker antagonized the pro-convulsive effect of AEA, suggesting that the potential mechanism underlying the effect of AEA depends on the CB_1_ receptor. It is possible that AEA aggravates seizures by suppressing GABA release from inhibitory presynaptic terminals (Lourenço et al., 2011).

As described above, AEA acts as a full agonist of TRPV1 (Smart et al., 2000). TRPV1 is a Ca^2+^-permeable cation channel, and its activation depolarizes neurons. TRPV1 activation is reported to suppress 2-AG synthesis and increase tonic inhibition through the reduction of tonic 2-AG signaling (Lee et al., 2015). Its activation also increases AMPA receptor endocytosis at excitatory postsynaptic sites and induces LTD (Chávez et al., 2010). The overall contribution of TRPV1 activation is considered pro-convulsive, as the TRPV1 agonist capsaicin was found to aggravate PTZ-induced seizures (Manna and Umathe, 2012). Furthermore, TRPV1 knockout mice exhibit decreased mortality due to PTZ-induced seizures (Jia et al., 2015). Therefore, AEA could be pro-convulsive through activation of TRPV1.

### Involvement of the CB_1_ Receptor

Early studies repeatedly demonstrated the suppressive effects of the CB_1_ agonists, Δ^9^-THC and WIN55212-2, on seizures (Boggan et al., 1973; Wada et al., 1975; Wallace et al., 2001). However, a protective effect of CB_1_ receptor signaling against acute seizures was clearly demonstrated later using a kainate-induced acute seizure model in CB_1_ knockout mice (Marsicano et al., 2003). A subsequent study demonstrated that CB_1_ receptors in hippocampal excitatory neurons, but not those in inhibitory neurons, were necessary to suppress kainate-induced seizures (Monory et al., 2006) despite the fact that the expression of CB_1_ mRNA in glutamatergic neurons were lower than in GABAergic interneurons (Marsicano and Lutz, 1999). Furthermore, overexpression of CB_1_ receptors in hilar mossy cells of the dentate gyrus and in pyramidal cells of the hippocampal CA1, CA2 and CA3 regions were shown to significantly reduce the severity of kainate-induced SE (Guggenhuber et al., 2010) and the expression of CB_1_ receptors in excitatory neurons of the cerebral cortex, hippocampus, and amygdala of global CB_1_ receptor knockout mice was sufficient to prevent the exacerbation of kainate-induced seizures in these mice (Ruehle et al., 2013). Taken together, these results indicate that CB_1_ receptor signaling at excitatory synaptic terminals in the hippocampus has a suppressive effect on kainate-induced seizures (Table 1).

In addition to kainate-induced seizures, endocannabinoid signaling mediated by CB_1_ receptors also effectively suppresses pilocarpine-induced seizures (Kow et al., 2014; Carletti et al., 2015). Recently, it was reported that the suppressive effect of WIN55212-2 on pilocarpine-induced seizures in rats was further enhanced by the co-administration of the 5-HT_2B/2C_ receptor agonist RO60-0175, which RO60-0175 application alone did not affect seizures (Colangeli et al., 2019). Furthermore, the 5-HT_2B_ receptor antagonist RS127445 significantly blocked the effects of WIN55212-2 and RO60-0175 co-administration. Therefore, CB_1_ and 5-HT_2B_ receptor signaling can synergistically suppress pilocarpine-induced seizures.

Seizures evoked by electrical stimulation of the amygdala were more severe in excitatory neuron-specific CB_1_ knockout mice and milder in inhibitory neuron-specific CB_1_ knockout mice than in wild-type mice (von Rüden et al., 2015b). These results are consistent with the notion that the activation of CB_1_ receptors at excitatory synapses suppress, whereas those at inhibitory synapses disinhibit and aggravate seizures (Table 1).

In marked contrast to seizures induced by kainate, pilocarpine, or electrical stimulation, the severity of PTZ-induced seizures is similar between CB_1_ knockout mice and their wild-type littermates (Naydenov et al., 2014). Some reports have shown both preventive (Naderi et al., 2011; Vilela et al., 2013) and promoting effects (Vilela et al., 2013) of CB_1_ receptor signaling on PTZ seizures. As of now, it is difficult to determine whether CB_1_ receptor signaling has anti- or pro-convulsive effects on PTZ-induced seizures and this issue requires further detailed investigation (Table 1).

The results described above were obtained from seizures in the non-epileptic brain. In rats with spontaneous seizures after pilocarpine-induced SE, the number of spontaneous seizures was significantly decreased by the CB_1_ receptor agonist WIN55212-2 and significantly increased by the CB_1_ receptor antagonist SR141716A (Wallace et al., 2003). Consistent results were obtained by using the CB_1_ receptor blocker AM251 for spontaneous seizures after kainate-induced SE (Sugaya et al., 2016) and by using positive allosteric modulator of CB_1_ receptor for the spontaneous absence seizures in GAERS rats (Roebuck et al., 2021). These results indicate that CB_1_ receptor signaling in the epileptic brain suppresses the occurrence of spontaneous seizures in the SE model and the absence seizure model (Figure 2 and Table 1).

WIN55212-2 injection decreased the number of spontaneous spike-and-wave discharges for 3 h in WAG/Rij rats. Interestingly, very long, sporadic trains of spike-and-wave discharges were observed in WAG/Rij rats from 3 h after WIN55212-2 injection (van Rijn et al., 2010), which is in sharp contrast to the decreased duration of spike-and-wave discharges after CB_1_ activation in GAERS rats (Roebuck et al., 2021). These long spike-and-wave discharge trains were partially suppressed by co-administration of AM251 (van Rijn et al., 2010), suggesting the involvement of CB_1_ receptor signaling in these abnormal paroxysmal discharges. Therefore, CB_1_ receptor signaling potentially decreases the number of absence seizures but increases the duration of each seizure in WAG/Rij rats.

### Involvement of the CB_2_ Receptor

With regard to acute seizure models, a single administration of AM630, a CB_2_ receptor antagonist, had no impact on seizures evoked by electrical stimulation of the perforant path in anesthetized rats (Rizzo et al., 2014) and by kainate injection in mice (Sugaya et al., 2016). However, when AM630 was administered to wild-type mice treated with AM251 or to CB_1_ receptor knockout mice, kainate-induced acute seizures were aggravated (Sugaya et al., 2016). These results suggest that CB_2_ receptor signaling may function as a protective backup in neural circuits when their excitability is excessive due to the disruption of CB_1_ receptor signaling. This notion is supported by the results of a study in which the effect of AM630 on evoked seizures was investigated in the epileptic brain. Animals that underwent repeated PTZ administration become highly susceptible to seizures, a process called PTZ kindling. Administration of the CB_2_ antagonist AM630 resulted in longer PTZ-induced seizures in fully PTZ-kindled rats (Aghaei et al., 2015). These results suggest that CB_2_ receptor signaling alleviates seizures when the excitability of neural circuits becomes so high that CB_2_ receptors are sufficiently activated (Figure 2 and Table 1). The anti-convulsive effect of CB_2_ receptor signaling has also been reported (Tchekalarova et al., 2018) by using mice in which seizures were induced by transcorneal electrical stimulation under the condition that the CB_2_ receptor was activated by its agonist β-caryophyllene (Gertsch et al., 2008; Tchekalarova et al., 2018).

In contrast, a pro-convulsant effect of CB_2_ receptor signaling has been reported in an acute PTZ (70 mg/kg, i.p.)-induced seizure model (de Carvalho et al., 2016). However, the seizures in the control group in this study were milder than those in previous reports of PTZ-induced seizures (Naydenov et al., 2014; Aghaei et al., 2015). It is therefore possible that CB_2_ receptor signaling might be pro-convulsive when the seizures are relatively mild. A recent study using a higher dose of PTZ (100 mg/kg, s.c.) reported increased seizure susceptibility in CB_2_ knockout mice (Shapiro et al., 2019). It is therefore possible that CB_2_ receptor signaling exerts a suppressive effect on ictogenesis in a brain state with relatively high excitability. Indeed, a recent study demonstrated a therapeutic potential of CB_2_ receptor-targeted positive allosteric modulators for epilepsy in animal models of electrical stimulation-induced seizures and PTZ-induced seizures as well as mice carrying the human *SCN1A* R1648H mutation associated with the condition known as genetic epilepsy with febrile seizures plus (Shapiro et al., 2021).

## Cannabidiol

In recent years, there has been much interest in the use of CBD for the treatment of epilepsy. Initially CBD (average 118 mg/kg, i.p.) was reported to have an anticonvulsant effect against seizures induced by transcorneal electrical stimulation in mice (Karler et al., 1973) and rats (Karler et al., 1974). A following study by the same research group showed that CBD had an antiepileptic effect without tolerance (Karler and Turkanis, 1980). Subsequently, many studies using various animal models of epilepsy have confirmed the anticonvulsant effects of CBD (Jones et al., 2010, 2012; Lima et al., 2020). However, the exact mechanisms by which CBD suppresses seizures remain unclear.

Cannabidiol has very low affinity for CB_1_ and CB_2_ receptors (Petitet et al., 1998; Jones et al., 2010) and acts as an inverse agonist (Thomas et al., 2007) or negative allosteric modulator of the CB_1_ receptor (Straiker et al., 2018). However, contrasting results have been reported recently showing a partial agonistic effect of CBD on CB_2_ receptor signaling in cultured cells (Tham et al., 2019). In line with this report, a very recent study using CB_1_ and CB_2_ knockout mice demonstrated the CB_2_ receptor signaling-dependent action of CBD on sucrose self-administration in mice (Bi et al., 2020)—CBD (10 to 40 mg/kg, i.p.) dose-dependently decreased sucrose consumption in wild-type and CB_1_ knockout mice, but not in CB_2_ knockout mice. Moreover, a CB_2_ antagonist, AM630, blocked the CBD-induced decrease in sucrose consumption in wild-type mice. These results demonstrate that CBD activates CB_2_ receptor signaling in the CNS. However, further studies are necessary to conclude that CBD does bind to the CB_2_ receptor and trigger its downstream signaling.

Cannabidiol is also reported to affect the degradation of endocannabinoids. CBD was shown to block the activity of FAAH (Bisogno et al., 2001) and increase the serum concentration of AEA as well as those of two additional FAAH substrates, palmitoylethanolamide and oleoylethanolamide, in human subjects (200 mg to 800 mg of CBD/day) (Leweke et al., 2012). Furthermore, injection of CBD (3 nmol) into the periaqueductal gray of rats increased the concentration of 2-AG in lipid extracts at the injection site to levels 2.6-fold higher than that of rats with vehicle injection (Maione et al., 2011). Therefore, CBD might suppress seizures by increasing the levels of AEA and/or 2-AG. Moreover, in agreement with these observations regarding increase in endocannabinoids levels, CBD (30 mg/kg, i.p.) delayed the latency to behavioral seizures and reduced their severity during pilocarpine-induced SE (Lima et al., 2020). The anticonvulsant effect of CBD is also reported to be blocked by pre-treatment with AM251, suggesting that CBD exerts its anticonvulsant effect through CB_1_ receptor signaling (Lima et al., 2020).

As mentioned earlier, endocannabinoids can act on GPR55 (Baker et al., 2006). CBD is reported to antagonize GPR55 signal and thereby increase the excitability of inhibitory interneurons (Kaplan et al., 2017). This effect might underlie the anticonvulsive effect of CBD (Kaplan et al., 2017). Also, CBD is reported to bind to and activate TRPV1 (Bisogno et al., 2001), which induces rapid desensitization of TRPV1 (Bisogno et al., 2001; De Petrocellis et al., 2011). Therefore, CBD might act as a blocker of TRPV1, although CBD-mediated increase in AEA may activate TRPV1. Thus, the overall effect of CBD on TRPV1 receptors is still unknown. Other effects of CBD include binding and activation of the 5-HT_1A_ receptor, which was demonstrated by using the [^35^S] GTPγS binding assay (Russo et al., 2005), and blockade of voltage-gated sodium channels (Patel et al., 2016; Sait et al., 2020). Because the activation of 5-HT_1A_ receptor ameliorates pilocarpine-induced SE (Clinckers et al., 2004) and the blockade of voltage-gated sodium channels is the major mechanism for the action of currently used anticonvulsants, CBD could suppress seizures through these pathways.

In addition to ictogenesis, CBD can potentially modulate epileptogenesis. CBD treatment (100 mg/kg, i.p.) during kainate-induced SE reduced the atrophy and death of parvalbumin- and cholecystokinin-expressing interneurons (Khan et al., 2018). Similarly, Lima et al. (2020) reported that CBD treatment (30 mg/kg, i.p.) prior to pilocarpine injection reduced the level of neuronal degeneration after pilocarpine-induced SE, while valproate treatment, which was used as a control for CBD treatment, did not. These studies suggest a protective effect of CBD against seizure-induced cell death. In Wister audiogenic rats, chronic CBD treatment (25 mg/kg, i.p., twice a day for 10 days) significantly suppressed the development of seizure responses in audiogenic kindling, indicating the antiepileptogenic effect of CBD in audiogenic kindling model (Lazarini-Lopes et al., 2021). Moreover, CBD treatment (200 mg/kg, orally administered) after the onset of spontaneous recurrent seizures in a pilocarpine-induced SE model not only suppressed seizure frequency but also ameliorated reference memory and working memory errors of epileptic mice in the hole-board task (Patra et al., 2019).

Clinical studies for testing the anticonvulsant effect of CBD in epileptic patients have been conducted since 1980 (Cunha et al., 1980). Recently, several significant clinical studies on CBD have been conducted in the form of randomized, double-blind, placebo-controlled trials. The results demonstrated that CBD was significantly more effective than placebo in the add-on treatment of convulsive seizures in Dravet syndrome at 10 and 20 mg/kg/day (Devinsky et al., 2017; Miller et al., 2020), drop seizures in Lennox-Gastaut syndrome at 10 and 20 mg/kg/day (Devinsky et al., 2018; Thiele et al., 2018), and focal and generalized seizures in tuberous sclerosis complex (TSC) at 25 and 50 mg/kg/day (Thiele et al., 2021). The percentage reduction in the number of convulsive or drop seizures compared to placebo was approximately 23–30% in patients with Dravet syndrome (Devinsky et al., 2017; Miller et al., 2020), 17–22% in patients with Lennox-Gastaut syndrome (Devinsky et al., 2018; Thiele et al., 2018), and 28.5–30.1% in patients with TSC (Thiele et al., 2021). Several open-label studies have also reported the suppressive effect of CBD on treatment-resistant epilepsy with various etiologies, including *CDKL5* mutation, childhood infection, and Ohtahara syndrome, in adults (Devinsky et al., 2016; Szaflarski et al., 2018) and children (Devinsky et al., 2016; Szaflarski et al., 2018; Park et al., 2020).

A meta-analysis regarding the adverse effects of CBD (Chesney et al., 2020) showed that CBD treatment was associated with abnormal results in liver function tests [odds ratio (OR): 11.19, 95% confidence interval (CI): 2.09–60.02], pneumonia (OR: 5.37, 95% CI: 1.17–24.65), decreased appetite (OR: 3.56, 95% CI: 1.94–6.53), diarrhea (OR: 2.61, 95% CI: 1.46–4.67), somnolence (OR: 2.23, 95% CI: 1.07–4.64), and sedation (OR: 4.21, 95% CI: 1.18–15.01). However, the analysis suggested that the abnormal results in liver function tests, somnolence, sedation, and pneumonia may have been caused by the interaction of CBD with other anticonvulsants. CBD inhibits several types of cytochrome P450 enzymes, which are necessary for the hepatic metabolism of other medications such as clobazam and sodium valproate (Zendulka et al., 2016). A subsequent meta-analysis revealed that the combination of CBD and clobazam increased the concentration of major active metabolites of clobazam, without affecting the concentration of CBD and clobazam (Patsalos et al., 2020). Thus, further studies are necessary about how CBD interacts with other medications.

## Summary and Future Perspectives

Endocannabinoid signaling is closely linked to the pathogenesis of epilepsy and is affected by a variety of factors. Generally, seizures cause an increase in the levels of endocannabinoids, especially 2-AG, which has an acute suppressive effect on seizures and can lead to chronic circuit changes. In particular, the transient surge in endocannabinoid signaling during the initial insult promotes epileptogenesis. Homeostatic downregulation of endocannabinoid signaling molecules, such as DGLα and the CB_1_ receptor, is likely to be induced by the increased production of endocannabinoids following initial injury; thus, prevention of epileptogenesis by DGLα or CB1 receptor antagonists immediately after the initial insult may be a potential therapeutic candidate. However, the role of endocannabinoid signaling changes from pro-epileptogenic to anti-epileptogenic during epileptogenesis or in epileptogenesis with milder progression such as kindling. Therefore, careful analyses of the changing roles of endocannabinoid signaling in epileptogenesis should be conducted in the future.

To develop new antiepileptic drugs and novel therapies for epilepsy, we need to understand the effects of endocannabinoids on ictogenesis as well as their mechanisms of action. Endocannabinoids mediate the retrograde suppression of synaptic transmission through presynaptic CB_1_ receptors—CB_1_ receptor signaling at excitatory synapses appears to have a suppressive effect, whereas that at inhibitory synapses has a promoting effect on seizures. In addition, 2-AG suppresses seizures through multiple downstream signaling pathways, including those involving the CB_1_, CB_2_, and GABA_A_ receptors. In contrast, AEA appears to have a minor role, if any, in suppressing seizures or even exert a promoting effect on ictogenesis due to its action on the TRPV1 receptor.

Three important issues remain unresolved. First, how is the contribution of CB_2_ receptor signaling to epileptogenesis and ictogenesis? Compared to the studies on the CB_1_ receptor, very few studies used conditional CB_2_ receptor knockout mice. Several experimental procedures for ictogenesis and epileptogenesis should be applied to conditional CB_2_ receptor knockout mice to determine the cell-types responsible for the role of CB_2_ receptors in ictogenesis and epileptogenesis. Moreover, since precise subcellular localization of CB_2_ receptors is not known, novel methods such as CRISPR-Cas9 mediated tagging of protein, SLENDR (Mikuni et al., 2016) and vSLENDR (Nishiyama et al., 2017), should be applied to the brains of normal and epileptic mice.

Second, how are the mechanisms underlying the increase in CB_1_ receptor expression during epileptogenesis? It is difficult to explain this change from the homeostatic point of view because the increase in CB_1_ receptor expression seems to occur predominantly at inhibitory synapses. This would lead to the reduction of inhibitory synaptic transmission and exaggerates the hyperexcitability of the epileptic circuit. As mentioned above, involvement of CB_1_ receptor signaling and that of interleukin-1β signaling seem to be critical to this process (Feng et al., 2016). Further studies are needed to clarify how these two molecular pathways interact with each other during epileptogenesis.

Third, how are the detailed time course and cellular origin of endocannabinoid release during epileptogenesis? Taking advantage of endocannabinoid imaging using GRABeCB2.0 (Dong et al., 2021), transient surges of 2-AG correlated with cellular activity have been observed during kainate-induced SE (Farrell et al., 2021). GRABeCB2.0 makes it possible to follow the changes in endocannabinoid release during epileptogenesis in the same animal, which is helpful for elucidating the mechanisms underlying the changes in the endocannabinoid system during epileptogenesis.

In conclusion, it is hoped that unraveling these issues will advance our understanding of the pathogenesis of epilepsy and will lead to the development of new antiepileptic drugs and disease-modifying therapies for epileptogenesis.

## Author Contributions

YS and MK designed and wrote the manuscript. Both authors contributed to the article and approved the submitted version.

## Conflict of Interest

The authors declare that the research was conducted in the absence of any commercial or financial relationships that could be construed as a potential conflict of interest.

## Publisher’s Note

All claims expressed in this article are solely those of the authors and do not necessarily represent those of their affiliated organizations, or those of the publisher, the editors and the reviewers. Any product that may be evaluated in this article, or claim that may be made by its manufacturer, is not guaranteed or endorsed by the publisher.
